# Identification of effective diagnostic biomarker and immune cell infiltration characteristics in acute liver failure by integrating bioinformatics analysis and machine-learning strategies

**DOI:** 10.3389/fgene.2022.1004912

**Published:** 2022-09-28

**Authors:** Mengqin Yuan, Lichao Yao, Xue Hu, Yingan Jiang, Lanjuan Li

**Affiliations:** ^1^ Department of Infectious Diseases, Renmin Hospital of Wuhan University, Wuhan, China; ^2^ State Key Laboratory for Diagnosis and Treatment of Infectious Diseases, National Clinical Research Centre for Infectious Diseases, Collaborative Innovation Centre for Diagnosis and Treatment of Infectious Diseases, The First Affiliated Hospital, Zhejiang University School of Medicine, Hangzhou, China

**Keywords:** acute liver failure, lasso logistic regression, SVM-RFE, diagnostic biomarker, immune cell infiltration

## Abstract

**Background:** To determine effective biomarkers for the diagnosis of acute liver failure (ALF) and explore the characteristics of the immune cell infiltration of ALF.

**Methods:** We analyzed the differentially expressed genes (DEGs) between ALF and control samples in GSE38941, GSE62029, GSE96851, GSE120652, and merged datasets. Co-expressed DEGs (co-DEGs) identified from the five datasets were analyzed for enrichment analysis. We further constructed a PPI network of co-DEGs using the STRING database. Then, we integrated the two kinds of machine-learning strategies to identify diagnostic biomarkers of top hub genes screened based on MCC and Degree methods. And the potential diagnostic performance of the biomarkers for ALF was estimated using the AUC values. Data from GSE14668, GSE74000, and GSE96851 databases was performed as external verification sets to validate the expression level of potential diagnostic biomarkers. Furthermore, we analyzed the difference in the protein level of diagnostic biomarkers between normal and ALF mice models. Finally, we used CIBERSORT to estimate relative infiltration levels of 22 immune cell subsets in ALF samples and further analyzed the relationships between the diagnostic biomarkers and infiltrated immune cells.

**Results:** A total of 200 co-DEGs were screened. Enrichment analyses depicted that they are highly enriched in metabolism and matrix collagen production-associated processes. The top 28 hub genes were obtained by integrating MCC and Degree methods. Then, the collagen type IV alpha 2 chain (COL4A2) was regarded as the diagnostic biomarker and showed excellent specificity and sensitivity. COL4A2 also showed a statistically significant difference and excellent diagnostic effectiveness in the verification set. In addition, there was a significant upregulation in the COL4A2 protein level in ALF mice models compared with the normal group. CIBERSORT analysis showed that activated CD4 T cells, plasma cells, macrophages, and monocytes may be implicated in the progress of ALF. In addition, COL4A2 showed different degrees of correlation with immune cells.

**Conclusion:** In conclusion, COL4A2 may be a diagnostic biomarker for ALF, and immune cell infiltration may have important implications for the occurrence and progression of ALF.

## Introduction

Acute liver failure (ALF) is a lethal systemic disorder marked by the massive necrosis of hepatocytes, leading to the rapid loss of hepatic function (Wang et al., 2014). The pathogenesis of ALF is complicated and not fully clarified yet. Studies have found that different etiologies such as virus, drug toxicity, autoimmunity, and ischemia can cause acute liver injury, which can progress rapidly to ALF or even death (Seto et al., 2012; Xiao et al., 2021).

Typically, the management of patients with ALF is challenging, because of its rapid progression, poor prognosis, and high in-hospital mortality rates (Bernal et al., 2013). So far, orthotopic liver transplantation and subsequent lifelong immunosuppressive therapy are the most effective methods for treating ALF (Yu et al., 2019; Li, 2021). However, the severe shortage of donor organs, high cost, and immunosuppression-related complications limited its practical applications (Karvellas et al., 2021; Yu et al., 2022). Therefore, identifying potential biomarkers before the deterioration of ALF is of great significance for survival rate improvement.

With the rapid advancement in high-throughput sequencing technology, bioinformatics analysis, and machine-learning strategy can be performed to identify novel diagnostic biomarkers for different clinical diseases (Picard et al., 2021). Tang et al. adopted novel feature selection strategies combined with a random forest (RF) algorithm to construct the classifiers that can identify the site of tumor origin with high specificity based on the DNA methylation profiles (Tang et al., 2018). Moreover, Yu et al. identified LSP1, GNLY, and MEOX2 may be diagnosis-related biomarkers of rheumatoid arthritis by integrating RF, least absolute shrinkage and selection operator (LASSO) logistic regression, weighted correlation network analysis (WGCNA), and support vector machine recursive feature elimination (SVM-RFE) algorithm (Yu et al., 2021). However, such studies that integrated bioinformatics analysis and machine-learning strategies to analyze the gene expression profile of ALF remain very rare. Furthermore, increasing research revealed that immune cells crucially participated in the incidence and development of ALF (Casulleras et al., 2020). Compared with acutely decompensated AF, patients with ACLF display increased leukocyte, neutrophil, and monocyte counts but accompanied by lymphopenia, which may contribute to immunosuppression in ACLF (Weiss et al., 2020). However, previous studies predominantly focused on the effect of individual immune cell types on the progression and prognosis of ALF. For example, CXCR1/CXCR2-expressing neutrophils in patients with ACLF may participate in hepatocyte death by direct contact and by the release of inflammatory mediators (Khanam et al., 2017). Therefore, a systematic method is urgently needed for clarifying the effect of different immune cells on the occurrence and progression of ALF.

In the present research, microarray datasets of healthy and ALF samples downloaded from the Gene Expression Omnibus (GEO) database were analyzed. For identifying the diagnostic biomarkers of ALF patients, we combined LASSO logistic regression and the SVM-RFE algorithm. The diagnostic efficacy of the potential diagnostic biomarkers was assessed according to the receiver operating characteristic (ROC) curve analysis. Moreover, we further assessed the association between the diagnostic biomarkers expression and infiltration of various immune cells.

## Materials and methods

### Overall study design

**FIGURE 1:**
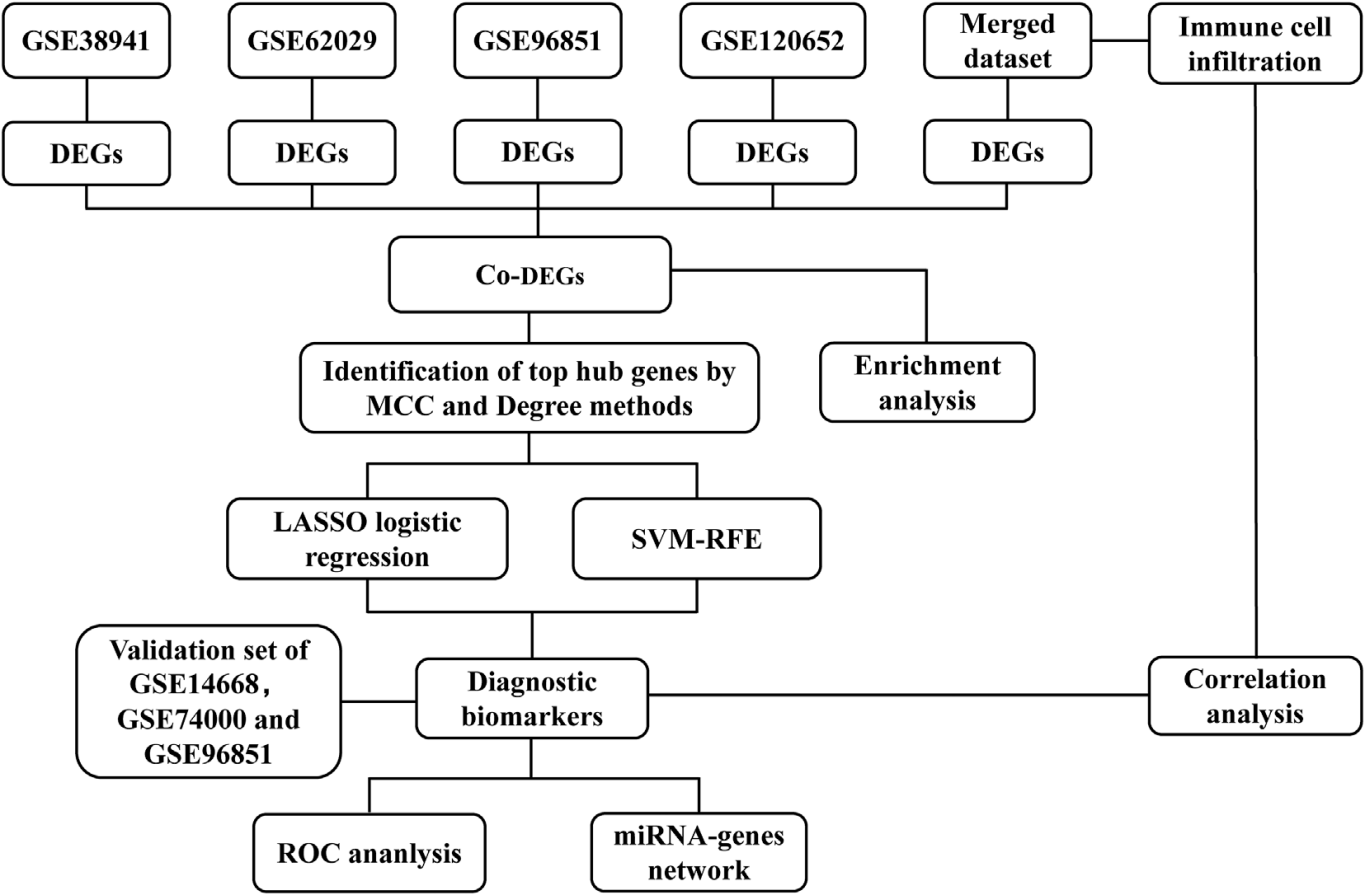
Flowchart of data collection and analysis.

The overall design and flow diagram of this study are shown in Figure 1. We first screened differentially expressed genes (DEGs) from five datasets. Based on the co‐expressed DEGs (co-DEGs) of the five datasets, we carried out enrichment analysis and identified the top 30 hub genes using the MCC and Degree methods. Subsequently, LASSO logistic regression and SVM-RFE algorithm were used to identify diagnostic biomarkers for ALF. Furthermore, ROC curve analyses were carried out to verify the potential diagnostic performance of the biomarkers for ALF in both the merged dataset and external validation dataset. We further verified the differential expression of diagnostic biomarkers between normal mice and ALF mice by performing Western blotting. Moreover, we adopted CIBERSORT to estimate relative infiltration levels of 22 immune cell subsets in ALF samples and further analyzed the relationship between immune cells and diagnostic biomarkers. Finally, to clarify the potential molecular mechanism of diagnostic biomarkers, we conducted a miRNA-genes interaction network analysis.

### Data collection

We collected gene expression profiling data (GSE38941, GSE62029, GSE96851, and GSE120652) from the GEO database (http://www.ncbi.nlm.nih.gov/geo/). GSE38941 data set includes liver samples of 17 ALF and 10 healthy controls. The GSE62029 data set includes liver samples of 13 ALF and 17 healthy controls. The GSE96851 data set includes liver samples of 17 ALF and 17 healthy controls. The GSE120652 data set includes liver samples of 3 ALF and 3 healthy controls. And the merged dataset consists of 50 ALF and 47 healthy controls from the four datasets mentioned above. Furthermore, 8 liver samples from ALF and 20 from healthy controls from GSE14668 combined with 3 liver samples from ALF and 2 from healthy controls from GSE74000 and 17 ALF and 17 healthy controls from GSE96851 were acted as validation set (Table 1).

**TABLE 1:** The samples from 7 datasets included in the study.

	GEO_ID	Normal	ALF	Platform
**Training set**	**GSE38941**	10	17	GPL570
	**GSE62029**	17	13	GPL570
	**GSE96851**	17	17	GPL570
	**GSE120652**	3	3	GPL6244
**Validation set**	**GSE14668**	20	8	GPL570
	**GSE74000**	2	3	GPL570
	**GSE96851**	17	17	GPL570

### Analysis of differential gene expression

DEGs between ALF and healthy groups in the five datasets were identified using the R package “Limma” (Ritchie et al., 2015). Those DEGs were defined as genes with expression differences of |log fold change (FC)| ≥ 1 and adjusted *p* value <0.05. R packages “pheatmap” and “ggplot2” were used to show the difference in the expression of DEGs. R package “VennDiagram” was used for screening co-DEGs of the five datasets (Chen and Boutros, 2011).

### Differential gene enrichment analysis

The Gene Ontology (GO) and Kyoto Encyclopedia of Genes and Genomes (KEGG) analyses based on these co-DEGs were performed using R package “clusterProfiler” (Yu et al., 2012). The significantly different GO terms and signal pathways were screened according to the threshold *p* value < 0.05 and q value < 0.05. Furthermore, Gene Set Enrichment Analysis (GSEA) software (version 4.1.0) was used to conduct GSEA. Enrichment analysis was considered to be statistically significant when FDR <0.25 and Nominal *p*-value < 0.05. Finally, the enrichment results of GSEA were displayed using R packages “ggplot2” and “grid” (Ito and Murphy, 2013).

### Protein-protein interaction (PPI) network construction

A PPI network of co-DEGs was constructed using the Search Tool for the Retrieval of Interacting Genes (STRING) database (https://string-db.org/) (Franceschini et al., 2013). The minimum interactive score was set to 0.70 of high confidence to ensure accuracy, and the strength of data support was indicated by line thickness. The top 30 hub genes were then identified using the MCC and Degree methods based on the CytoHubba plug-in of Cytoscape software (version 3.8.2) (Smoot et al., 2011). Then, the intersection of these hub genes was used for further research.

### Identification and verification of diagnostic biomarkers

We further identified diagnostic biomarkers by using LASSO logistic regression and SVM-RFE algorithm based on the hub genes screened from the five datasets. LASSO logistic regression was performed by the R package “glmnet” and minimal lambda was considered optimal. SVM-RFE algorithm was carried out using R package “e1071” with five-fold cross-validation (Engebretsen and Bohlin, 2019). Subsequently, we selected the overlapping genes for further analysis. To assess the diagnostic effectiveness of these genes, the R package “pROC” was used to calculate the area under the curve (AUC) values (Robin et al., 2011). We also compared the expression levels of the biomarkers between ALF and healthy groups and calculated the AUC value in the validation set.

### ALF mice model preparation

Six male C57BL/6 mice (5–6 weeks) were randomized to the ALF group and six to the control group. The mice were intraperitoneally injected with LPS (100 μg/kg) and d-GalN (400 mg/kg) to construct the ALF mice model. The normal control group was treated with the same volume of normal saline. The mice were sacrificed 24 h after inducing ALF, and liver samples were harvested for subsequent experimental analysis.

### Western blotting

The RIPA Lysis Buffer was performed to extract the total protein of liver tissues. The protein concentration was quantified using a BCA protein assay kit (Beyotime, China). Total protein of 20 μg per sample was resolved *via* SDS-polyacrylamide gel electrophoresis for 2 h and transferred onto PVDF membranes. The membrane was then blocked with 5% nonfat dried milk in Tris-buffered saline containing Tween-20 for 1 h. After that, the membrane was incubated overnight with primary antibodies at 4°C: collagen type IV alpha 2 chain (COL4A2) (A7657, 1:1000, ABclonal), GAPDH (ab9485, 1:1000, Abcam). Subsequently, membranes were incubated with HRP-conjugated goat anti-rabbit secondary antibodies at room temperature for 1 h. The protein bands were visualized by ECL reagent and then quantified by Image Lab software (version 4.1, Bio-Rad Laboratories, Inc.).

### Analysis of immune cell infiltration

Based on standardized gene expression profiles of 50 ALF liver samples and 47 healthy control liver samples from the merged dataset, the CIBERSORT algorithm was applied to speculate the relative fractions of 22 subtypes of infiltrated immune cells. CIBERSORT algorithm can transform the normalized gene expression matrix into the relative composition of 22 subtypes of infiltrated immune cells. We used the Wilcoxon test at *p* < 0.05 to identify the significant differences of significant infiltrating immune cells between ALF and control liver specimens. R packages “ggplot2”, “pheatmap” and “vioplot” were applied to visualize the differences in immune cell infiltration between ALF and healthy control liver samples. R package “corrplot” were used to visualize the correlation between individual immune cell subsets. Moreover, the correlation was analyzed to verify the association between the biomarkers and immune infiltration by using Spearman correlation analysis.

### Construction of miRNA-genes interaction network

We further used miRWalk (http://mirwalk.umm.uni-heidelberg.de/) database to predict miRNAs targeting these potential diagnostic biomarkers. And TargetScan (http://www.targetscan.org/vert_72/) and miRDB (http://www.mirdb.org/) databases were utilized for intersection operation. The criterion for selection was set at *p* < 0.05 and the length of the minimum seed sequence was 7 mer, and the binding region of the target gene was 3′UTR. Then, we used Cytoscape software to visualize the final result of the miRNA-genes interaction network.

## Results

### Identification of DEGs between ALF and control samples

**FIGURE 2:**
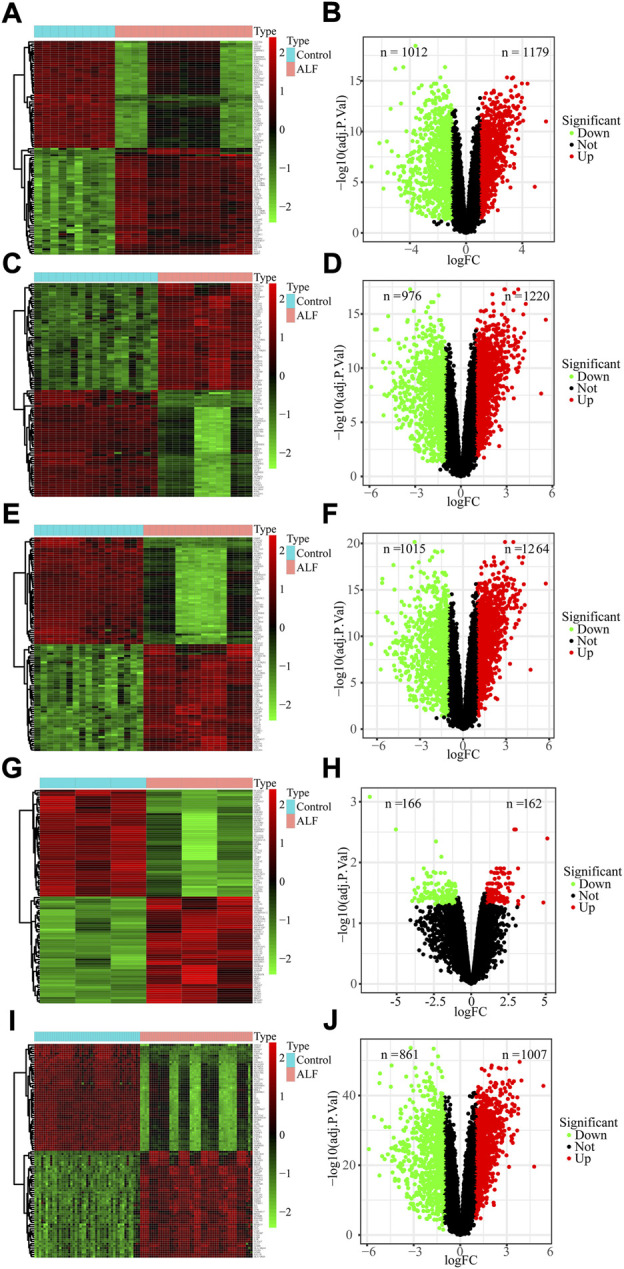
Identification of DEGs from GEO datasets. **(A,B)** GSE38941, **(C,D)** GSE62029, **(E,F)** GSE96851, **(G,H)** GSE120652 and **(I,J)** merged dataset.

**FIGURE 3:**
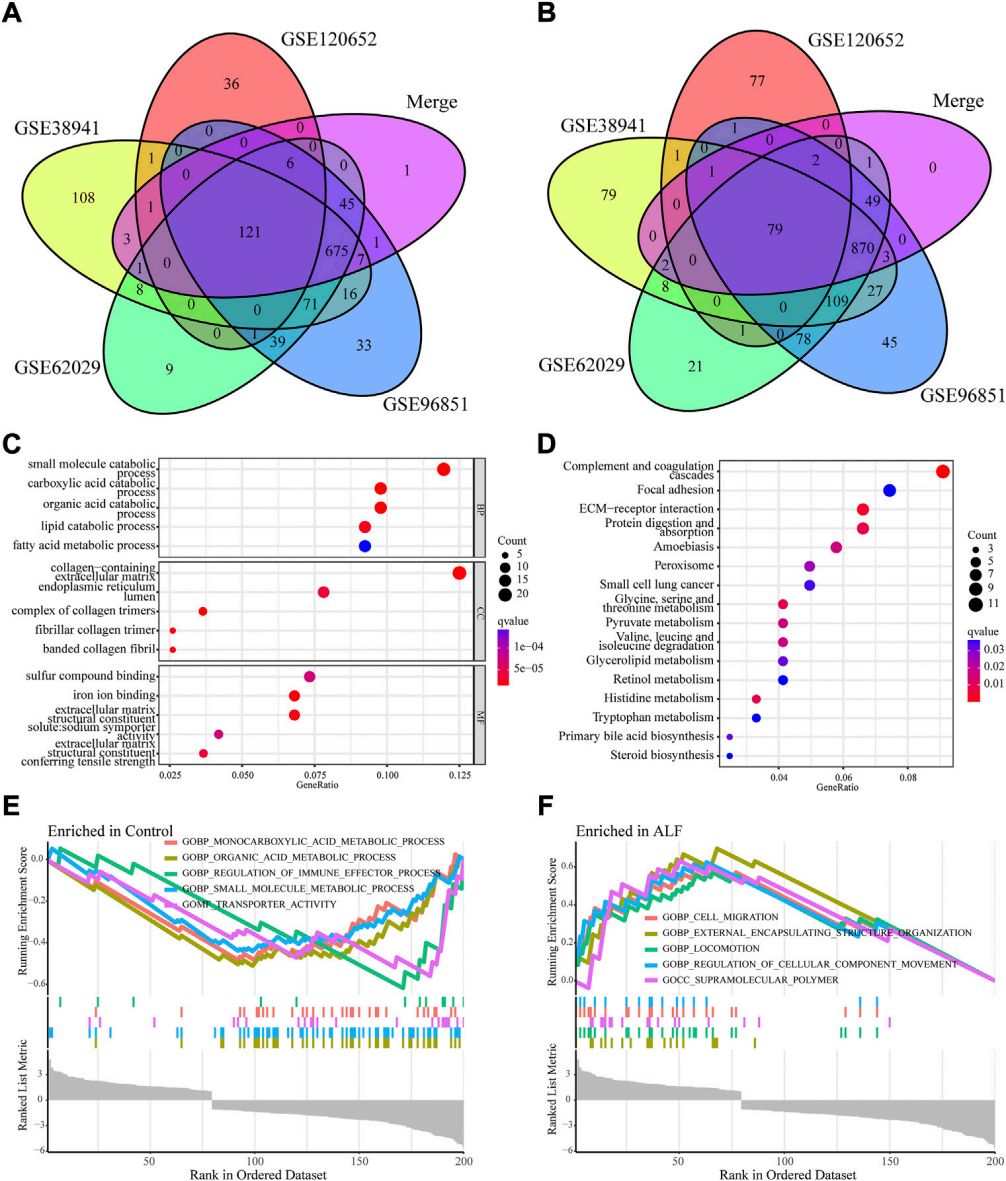
Function and pathway enrichment analysis of co-DEGs. **(A)** Venn diagram to identify co-downregulated DEGs between normal and ALF. **(B)** Venn diagram to identify co-upregulated DEGs between normal and ALF. **(C)** GO analysis of Co-DEGs. **(D)** KEGG analysis of Co-DEGs. **(E,F)** GSEA analysis of all genes.

To identify ALF-related genes, we screened the DEGs between ALF and normal controls in the five datasets. A total of 2191 DEGs were screened in the GSE38941 dataset, which consisted of 1179 genes up-regulated and 1012 genes down-regulated (Figures 2A,B, Supplementary Table S1). In the GSE62029 dataset, 1220 genes up-regulated and 976 genes down‐regulated were identified (Figures 2C,D). In the GSE96851 dataset, 1264 up-regulated and 1015 down-regulated genes were screened (Figures 2E,F). In the GSE120652 dataset, 162 up-regulated and 166 down-regulated genes were identified (Figures 2G,H). And in the merged dataset, we screened 1007 up-regulated and 861 down-regulated genes (Figures 2I,J). After removing these duplicate genes and genes with missing values, Venn maps were created by using co-downregulated and co-upregulated DEGs in GSE38941, GSE62029, GSE96851, GSE120652, and the merged dataset (Figures 3A,B). Finally, we identified 121 co-down-regulated and 79 co-up-regulated DEGs (Supplementary Table S2).

### Enrichment analyses of Co-DEGs

We applied enrichment analyses, including GO, KEGG, and GSEA, to study the biological functions of co‐DEGs. Figure 3C showed the top 12 GO terms according to the *p*-value. Co-DEGs are mainly involved in metabolism and matrix collagen production-related processes, including carboxylic acid catabolic process, small molecule catabolic process, lipid catabolic process, organic acid catabolic process, fatty acid metabolic process, collagen-containing extracellular matrix, extracellular matrix structural constituent, complex of collagen trimers, and extracellular matrix structural constituent conferring tensile strength. The top 16 KEGG terms of the co-DEGs are shown in Figure 3D. These co-DEGs mainly participated in complement and coagulation cascades, steroid biosynthesis, tryptophan metabolism, primary bile acid biosynthesis, histidine metabolism, ECM-receptor interaction, and focal adhesion. To further reveal the molecular mechanism associated with ALF, GSEA was executed based on the combining expression profiles of the training set. Figures 3E,F showed the significant enrichment of KEGG pathway in control group and ALF group. These KEGG pathways include monocarboxylic acid metabolic process, organic acid metabolic process, regulation of immune effector process, small molecule metabolic process, transporter activity, cell migration, external encapsulating structure organization, locomotion, regulation of cellular component movement, and supramolecular polymer.

### Identification of the top hub genes based on PPI network

**FIGURE 4:**
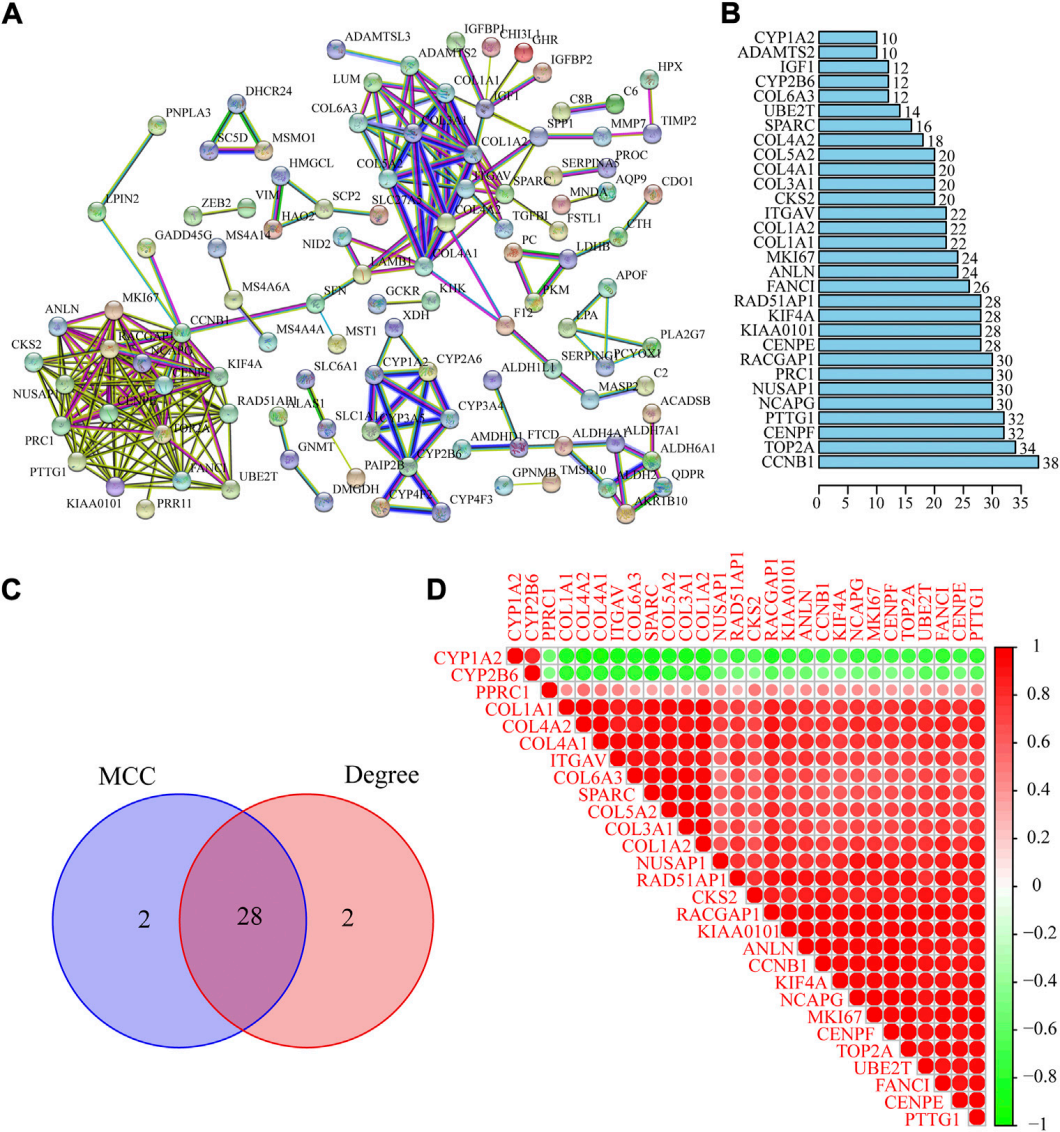
Identification and analysis of the PPI network of DEGs and hub genes. **(A)** PPI network was constructed using STRING database. **(B)** Top thirty gene in the PPI network. **(C)** Venn diagram to identify intersected hub genes screened by the MCC and Degree methods. **(D)** Pearson’s correlation analysis of 28 hub genes.

A PPI network of co-DEGs was built by using the STRING database. There are 197 nodes (genes) and 247 edges (the connection between nodes) contained in the PPI network (Figure 4A). The statistics of PPI network nodes of the top 30 genes are depicted in Figure 4B. Next, we used the Cytoscape plug-in “Cytohubba” to filter the top 30 hub genes of ALF based on the PPI network (Table 2). By taking the intersection of the 60 hub genes screened by the MCC and Degree methods respectively, 28 hub genes were identified (Figure 4C). The result of Pearson’s correlation analysis was depicted in Figure 4D. The result showed that CYP1A2 and CYP2B6 were negatively related to the other 26 hub genes.

**TABLE 2:** Top 30 hub genes screened by MCC and Degree methods.

Catelogy	MCC method			Degree method		
Geen symbol of top 30	SPARC	PTTG1	COL1A2	SPARC	MKI67	COL1A2
	CKS2	MKI67	COL5A2	CKS2	NUSAP1	COL5A2
	ANLN	NUSAP1	UBE2T	ANLN	PRC1	IGF1
	RAD51AP1	PRC1	FANCI	RAD51AP1	KIF4A	UBE2T
	CYP1A2	KIF4A	COL4A2	CYP1A2	CENPF	FANCI
	NCAPG	CENPF	KIAA0101	NCAPG	TOP2A	COL4A2
	CYP2A6	TOP2A	COL4A1	CCNB1	RACGAP1	KIAA0101
	CCNB1	RACGAP1	COL6A3	CYP2B6	COL1A1	COL4A1
	CYP2B6	COL1A1	ITGAV	CENPE	LAMB1	COL6A3
	CENPE	COL3A1	LUM	PTTG1	COL3A1	ITGAV

### Exploring candidate diagnostic biomarkers associated with ALF by LASSO regression and SVM-RFE

**FIGURE 5:**
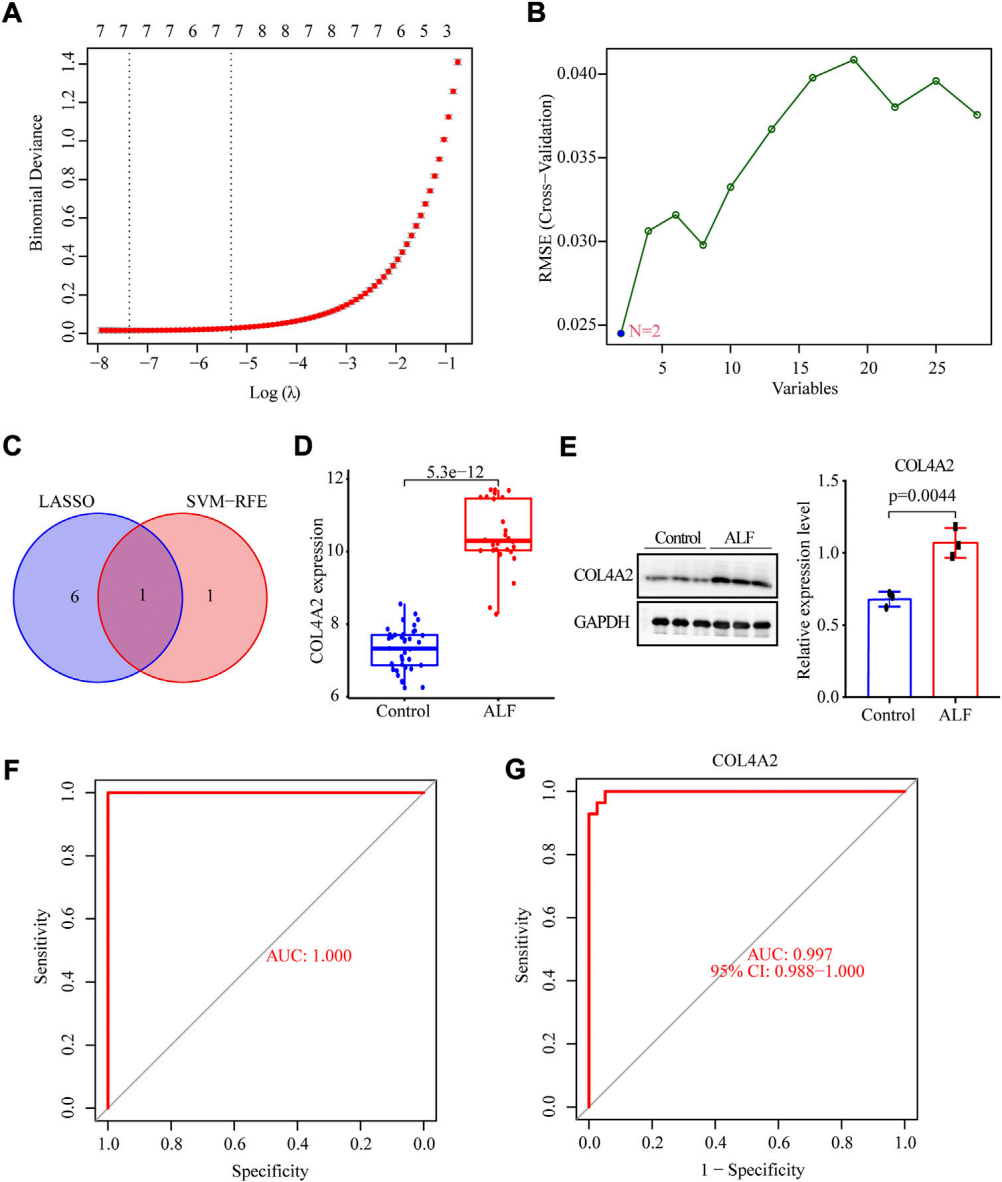
Identification of diagnostic biomarker of ALF *via* the comprehensive strategy. **(A)** LASSO logistic regression algorithm to screen diagnostic markers. **(B)** SVM-RFE algorithm to screen biomarkers. **(C)** Venn diagram to identify intersected DIAGNOSTIC biomarkers screened by the LASSO and SVM-RFE. **(D)** The expression level of COL4A2 in the validation set. **(E)** The protein levels of COL4A2 in normal and ALF mice models. The ROC curve of the gene signature in the testing set **(F)** and validation set **(G)**.

To identify which of these hub genes could be biomarkers for ALF diagnosis, we further analyzed the top 28 hub genes using the LASSO regression and SVM-RFE algorithm. A total of seven genes (CYP1A2, CYP2B6, PTTG1, COL3A1, UBE2T, COL4A2, and COL6A3) were identified by LASSO logistic regression algorithm, and two genes (SPARC, COL4A2) were identified as potential biomarkers by the SVM-RFE algorithm (Figures 5A,B). Finally, COL4A2 was an overlapping gene that was identified as a diagnostic biomarker (Figure 5C). The expression of COL4A2 in ALF was also significantly upregulated compared to control samples in the validation set (Figure 5D). Furthermore, we also verified the differential expression of diagnostic biomarkers between normal mice and ALF mice using Western blotting. The protein level of COL4A2 in the ALF model group was also obviously increased compared to the normal group (Figure 5E). ROC curve analysis was employed to assess the diagnostic effectiveness of COL4A2 for ALF in the merged dataset and validation set. The AUC values were respectively 1.00 and 0.997, indicating that COL4A2 had an excellent diagnostic capability to distinguish ALF from normal controls (Figures 5F,G).

### Analysis of immune cell infiltration

**FIGURE 6:**
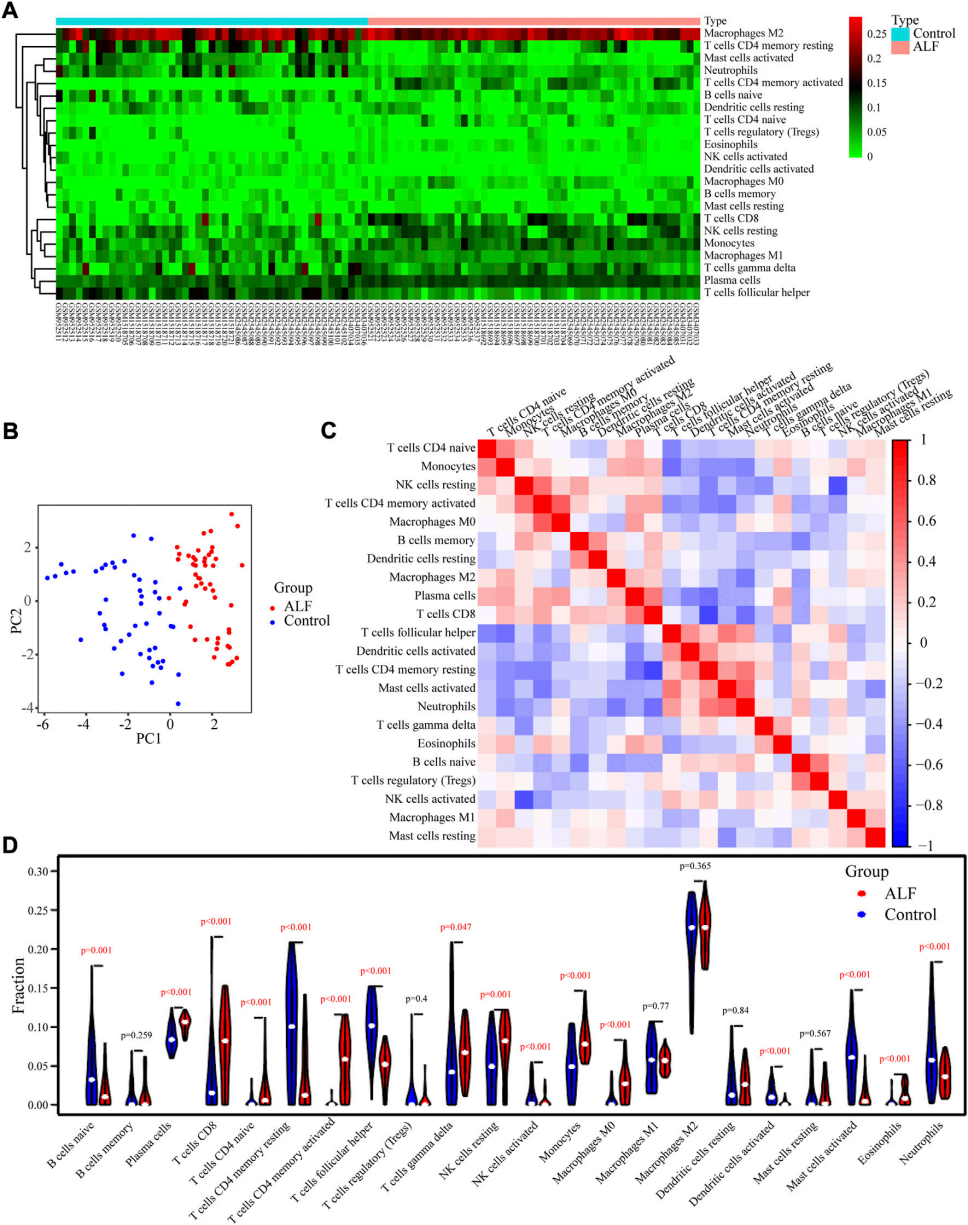
Characteristics of infiltrating immune cells. **(A)** Proportions of 22 immune cell subpopulations in normal and tissues. **(B)** PCA showed that 22 types of immune cells could roughly distinguish between and normal tissues. **(C)** Correlation coefficient heat map visualizing the interactions among immune cells. **(D)** Violin plot showing the immune cells with differential infiltration (*p* < 0.05).

The relevant proportions of 22 immune cells on the 50 ALF and 47 control samples of the training set were further studied using the CIBERSORT algorithm (Figure 6A). According to principal component analysis (PCA), there was a difference in patterns between the ALF and control groups. The result of PCA analysis revealed that there was a difference in immune infiltration status between ALF and control samples (Figure 6B). The heatmap of Pearson’s correlation showed the landscape of 22 infiltrating immune cells. M0 macrophages, plasma cells, resting NK cells, CD8 T cells, eosinophils, and monocytes had a positive correlation with activated CD4 memory T cells. While monocytes, CD8 T cells, plasma cells, activated CD4 memory T cells, and resting NK cells had a significantly inversely correlated with resting CD4 memory T cells (Figure 6C). The violin plot showed that CD8 T cells, plasma cells, gamma delta T cells, activated CD4 memory T cells, naive CD4 T cells, monocytes, resting NK cells, macrophages M0, and eosinophils infiltrated more in the ALF than the normal sample, while naive B cells, follicular T helper cells, resting CD4 memory T cells, activated NK cells, activated Dendritic cells, activated Mast cells, and neutrophils denoted the opposite (Figure 6D).

### Correlation analysis of COL4A2 and the immune cell infiltration

**FIGURE 7:**
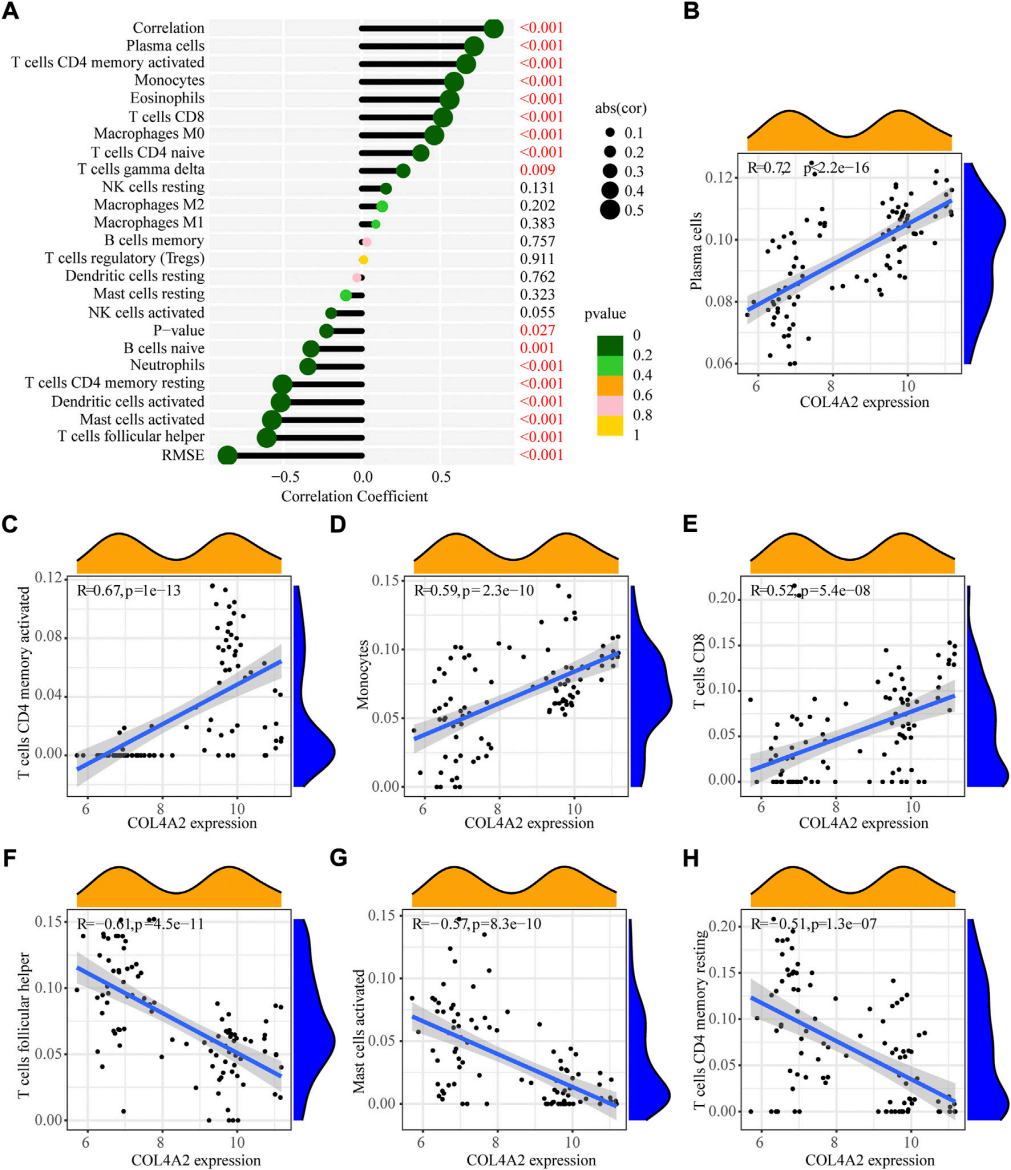
Pearson correlation of immune infiltrating cells with the signature genes. **(A)** Correlation between COL4A2 and infiltrating immune cells. **(B)** Plasma cells. **(C)** Activated CD4 memory T cells. **(D)** Monocytes. **(E)** CD8 T cells. **(F)** Follicular T helper cells. **(G)** Activated Mast cells. **(H)** Resting CD4 memory T cells.

Furthermore, correlation analysis (Figure 7) revealed that positively correlation between COL4A2 and plasma cells (R = 0.72, *p* < 2.2e−16), activated CD4 memory T cells (R = 0.67, *P* = 1e−13), monocytes (R = 0.59, *p* = 2.3e−10) and CD8 T cells (R = 0.52, *p* = 5.4e−08), while negatively correlation between COL4A2 and follicular T helper cells (R = −0.61, *p* = 4.5e−11), activated mast cells (R = - 0.57, *p* = 8.3e−10) and resting CD4 memory T cells (R = - 0.51, *p* = 1.3e−07).

### Further miRNA mining and interaction network analysis

**FIGURE 8:**
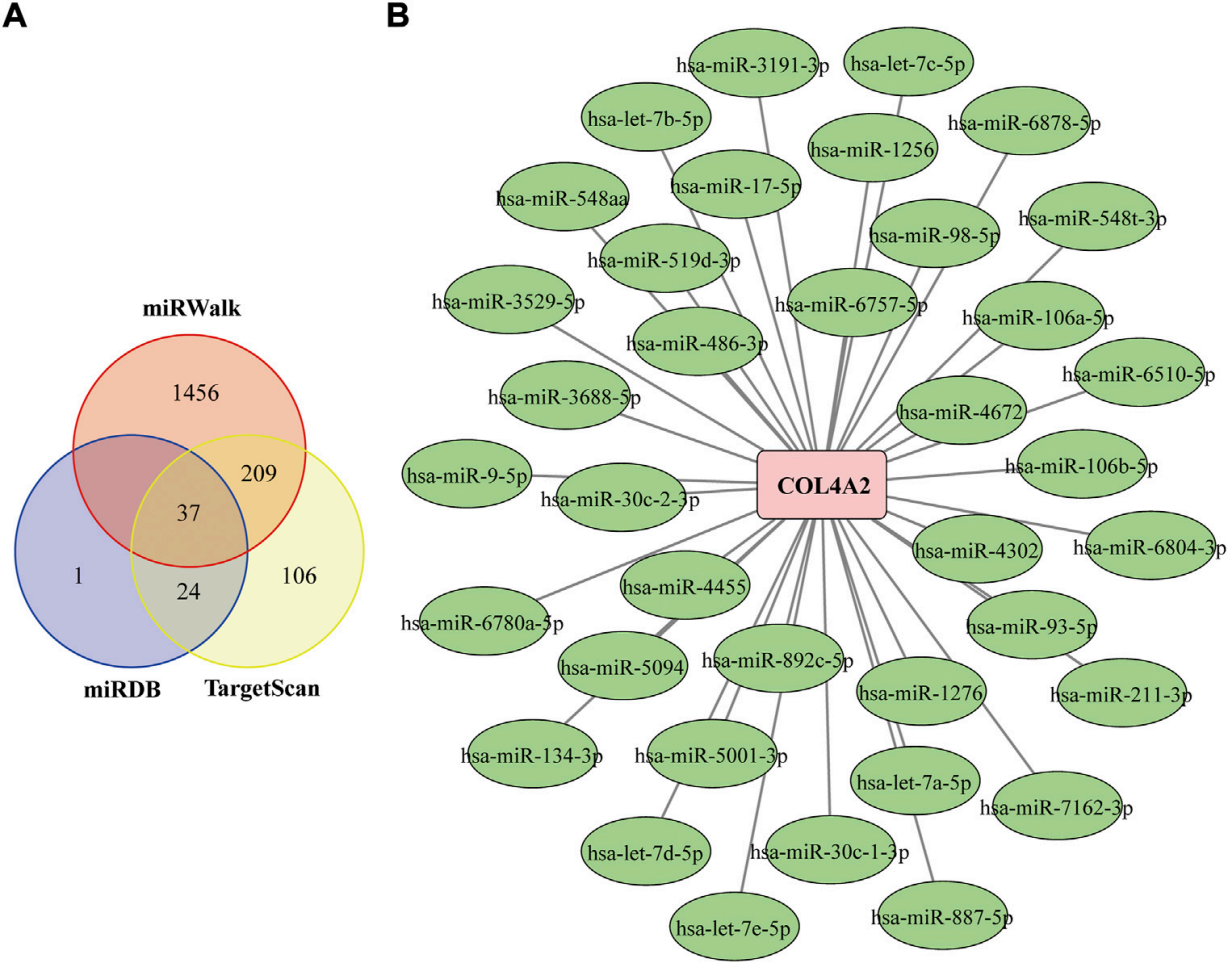
Construction of the COL4A2–miRNA Regulatory Network **(A)** Venn diagram of the intersection of targeted miRNAs screened by miRWalk, miRDB and TargetScan databases. **(B)** COL4A2–miRNA regulatory network. The square represents gene, and the circle represents miRNA. The green frame represents down-regulated miRNA, and pink frame means up-regulated gene.

MiRWalk, TargetScan, and miRDB databases were used to screen the potential miRNA that targeting COL4A2 (Fan et al., 2016). Select the intersection of miRNA results predicted by both miRWalk, TargetScan, and miRDB database as the final result. Finally, a total of 37 miRNAs were identified (Figure 8A). The miRNA-genes interaction network was shown in Figure 8B.

## Discussion

ALF is a life-threatening end-stage liver disease and is a medical emergency with considerable morbidity and mortality (Arroyo et al., 2020). In the absence of reliable and predictive biomarkers for the early diagnosis of ALF, patients often lose the best treatment opportunities and eventually leading to a poor prognosis. In this present research, we tend to identify diagnostic biomarkers for ALF and assess the effects exerted by the infiltrating immune cells in ALF.

We collected and analyzed ALF and healthy control samples from five mRNA microarray data sets (GSE38941, GSE62029, GSE96851, GSE120652, and the merged dataset). A total of 200 co-DEGs were identified from the training set, which consisted of 121 co-down-regulated and 79 co-up-regulated DEGs. Enrichment analysis indicated that these co-DEGs were highly involved in metabolism and matrix collagen production-related processes. Then, we selected 28 genes as hub genes of ALF from 200 co-DEGs and further identified COL4A2 as a potential diagnostic biomarker based on the LASSO logistic regression and SVM-RFE. COL4A2 is an important component in the extracellular matrix (Chuang et al., 2014). Previous studies have found that COL4A2 may be a tumor biomarker that promotes tumor metastasis and proliferation, which is highly expressed in liver preneoplastic lesions, such as fibrosis and cirrhosis (Chen et al., 2014; Dang et al., 2019). However, the significance of COL4A2 in the progression and prognosis of ALF has not been investigated yet. In our study, the AUC values were validated in the merged dataset and validation set, suggesting that COL4A2 had a high accuracy of predictive value for ALF. These results depicted that COL4A2 might be correlated with ALF development. However, further functional experiments are required to clarify the role of COL4A2 in ALF.

To further investigate the effect of immune cell infiltration, we applied CIBERSORT to reveal the relative proportions of 22 infiltrating immune cells of ALF. Our results depicted that there was low infiltration of naive B cells, while there were high infiltrations of CD8 T cells, plasma cells, activated CD4 memory T cells, naive CD4 T cells, gamma delta T cells, and monocytes, which was concordant with those in previous findings (Antoniades et al., 2008; Chen et al., 2019; Khanam and Kottilil, 2020). Previous work showed that B cell immunity is important in the progression of ALF, which was mainly mediated by a large number of intrahepatic IgG and IgM produced by plasma cells against the HBcAg (Farci et al., 2010). Consensus is growing that the unbalance of T cell subsets has been implicated in the occurrence and course development of ALF (Shen et al., 2020). Studies have indicated that activated CD4 T cells (such as Th17 cells and Treg cells) secrete a large number of proinflammatory cytokines, which leads to the amplification of systemic inflammatory response, while excessive systemic inflammatory response will lead to the further development of ALF (Dong et al., 2013). During the early stage of ALF, ligation of damage-associated molecular patterns (DAMPs) and pathogen-associated molecular patterns (PAMPs) to pattern-recognition receptors (PRRs) results in the activation of monocytes, and then monocytes initiate a systemic inflammatory response by releasing cytokines and chemokines (Triantafyllou et al., 2018; Casulleras et al., 2020). Furthermore, activated monocytes can also differentiate into macrophages. The chemokines, pro-inflammatory cytokines, and ROS released by the activated macrophages can further amplify the pro-inflammatory signal and promote the accumulation of other inflammatory cells in the liver to accelerate the development of a systemic inflammatory response (Possamai et al., 2014). The results of correlation analysis revealed that plasma cells and CD8 T cells are closely associated with the infiltration of activated CD4 memory T cells. Moreover, the Spearman correlation test indicated that the COL4A2 was significantly associated with plasma cells, activated CD4 T cells, CD8 T cells, and monocytes.

This study has a few limitations that need to be considered during interpreting our findings. The findings of our research were based on the public database and the small size of clinical samples, we do need to verify the robustness of the results of the study in basic research and clinical studies with larger sample sizes in the future. As the lack of clinical information, we did not explore the impact of COL4A2 on the outcomes of ALF patients. Furthermore, we only discussed the role of coding genes biomarkers in the diagnosis of ALF. Considering the recent trend of developing computational models for identifying non-coding RNA-related biomarkers of human complex diseases, non-coding RNA may have certain potential in the diagnosis of ALF, which is our future research direction (Zeng et al., 2018; Zeng et al., 2020).

## Conclusion

In conclusion, through the comprehensive analysis of GEO datasets by combining bioinformatics analysis and machine learning strategies, we found that COL4A2 may be a potential diagnostic biomarker for ALF. Further research is necessary to fully explore the precise role of COL4A2 in the pathogenesis of ALF. Besides, immune cell infiltration may play a critical function in the occurrence and development of ALF.

## Data Availability

The datasets presented in this study can be found in online repositories. The names of the repository/repositories and accession number(s) can be found in the article/Supplementary Material.
